# Simple graph models of information spread in finite populations

**DOI:** 10.1098/rsos.150028

**Published:** 2015-05-20

**Authors:** Burton Voorhees, Bergerud Ryder

**Affiliations:** 1 Center for Science, Athabasca University, 1 University Drive, Athabasca, Alberta, Canada T9S 3A3; 2 Department of Mathematics, University of Victoria, Victoria, British Columbia, Canada

**Keywords:** graph models of populations, fixation probabilities, graph parameters

## Abstract

We consider several classes of simple graphs as potential models for information diffusion in a structured population. These include biases cycles, dual circular flows, partial bipartite graphs and what we call ‘single-link’ graphs. In addition to fixation probabilities, we study structure parameters for these graphs, including eigenvalues of the Laplacian, conductances, communicability and expected hitting times. In several cases, values of these parameters are related, most strongly so for partial bipartite graphs. A measure of directional bias in cycles and circular flows arises from the non-zero eigenvalues of the antisymmetric part of the Laplacian and another measure is found for cycles as the value of the transition probability for which hitting times going in either direction of the cycle are equal. A generalization of circular flow graphs is used to illustrate the possibility of tuning edge weights to match pre-specified values for graph parameters; in particular, we show that generalizations of circular flows can be tuned to have fixation probabilities equal to the Moran probability for a complete graph by tuning vertex temperature profiles. Finally, single-link graphs are introduced as an example of a graph involving a bottleneck in the connection between two components and these are compared to the partial bipartite graphs.

## 2. Introduction

Using graphs to represent structured populations has become an important tool in approaching a variety of questions relating to the way that information spreads in non-homogeneous populations. Questions that have been addressed include the extinction or fixation of mutant genes [1–4]; epidemics [5–7]; spread of computer viruses, rumours and gossip [8–11]; development of rumour spreading algorithms for synchronization processes in parallel computation [12–14]; uptake of innovations and new ideas [15]; locating the source of rumours or viruses (natural and computer) [16,17]; and tracking terrorists [18,19].

A population is represented as a directed graph, each vertex corresponding to an individual population member, while edges are labelled with information describing the interaction between population members. In many cases, the edge labels are probabilities so that the weight *w*_*ij*_ of the edge that connects vertex *i* to vertex *j* describes the probability of an effect being propagated from vertex *i* to vertex *j*.

Following this, an update procedure is defined describing temporal dynamics. Here attention is restricted to discrete processes but continuous models have also been explored [20]. Some of the useful update procedures are birth–death, death–birth, voter models and probabilistic voter models [21]. In this paper, birth–death updating is used. In cases of rumour spread, there are competing narratives and birth–death processes provide a good model.

The state space approach developed in Voorhees [22] and Voorhees & Murray [23] allows determination of all fixation probabilities for arbitrary initial conditions. The population is partitioned into two disjoint classes, depending on the presence or absence of a defining characteristic (e.g. a mutant gene, believing a rumour, being infected with a virus, etc.). Vertices are labelled 0 if this characteristic is not present and 1 if it is present. Thus, for a population of fixed size *N* the state space is the set of 2^*N*^ binary vectors of length *N* with the all zero vector corresponding to extinction and the all one vector to fixation. A state vector ***v***=(*v*_1_,…*v*_*N*_) is also a binary number. Taking *v* as the denary form of this number, $v=\sum_{k=1}^{N}v_{k}2^{N-k}$, and denoting the probability that the state denoted *v* will go to fixation by *x*_*v*_, a set of master equations were derived [22], yielding fixation probabilities for all initial states.

2.1 $$[N+(r-1)m-r\text{a}(\text{v})\cdot \text{v}-\text{b}(\text{v})\cdot {\text{v}}^{\prime }]x_{v}-r\sum_{i=1}^{N}a_{i}(\text{v})v_{i}^{\prime }x_{v+2^{N-i}}-\sum_{i=1}^{N}b_{i}(\text{v})v_{i}x_{v-2^{N-i}}=0,$$

where *W* is the edge weight matrix of the population graph, *v*′_*i*_=1−*v*_*i*_ and ***a***(***v***)=***v***⋅*W*,***b***(***v***)=***v***′⋅*W*.

This yields interesting results [22,23] but is limited in that equations (2.1) are a set of 2^*N*^−2 linear equations in an equal number of unknowns so that exact solution for populations having more than a few members is not possible unless strong symmetry constraints are imposed.

As far as general solutions for fixation probabilities are concerned, Moran [24] derived the single vertex fixation probability for a complete graph on *N* vertices as

2.2 $$\rho_{M}=\frac{1-1/r}{1-1/r^{N}}=\frac{r^{N-1}}{1+r+\cdots +r^{N-1}},$$

and the *k*-vertex fixation probability for the complete graph is

2.3 $$\rho_{M,k}=\frac{r^{N-1}+\cdots +r^{N-k}}{1+r+\cdots +r^{N-1}}.$$

A graph is fixation equivalent to a complete graph if its single vertex fixation probability is given by the Moran expression of equation (2.2). Lieberman *et al.* [4] proved that a graph with a probabilistic edge weight matrix is fixation equivalent to a complete graph if and only if the edge weight matrix is doubly stochastic. Broom & Rychtàr [25] derived the single vertex fixation probability for the n-star graph and also provided a means to compute this probability for a line graph. Zhang *et al.* [26] derived the *k*-vertex fixation probability for star graphs, and Voorhees & Murray [23] give the single vertex fixation probability for the complete bipartite graph *K*_*s*,*n*_ together with summation formulae allowing computation of *k*-vertex fixation probabilities for these graphs. The star and bipartite results are reproduced in simple and compact form in Monk *et al.* [27] using a Martingale formalism, while results reported in Adlam & Nowak [28] indicate that the Moran formula of equation (2.1) may be, at least asymptotically, universal in the family of random graphs. In this paper, several classes of simple graphs are considered with respect to fixation probability and characteristic measures of graph structure.

Because applied interest is in large populations, a good deal of research is focused on random graphs, complete graphs and small world graphs with vertex degrees specified in terms of global averages and treated statistically [29–32]. Our goal is more modest, to provide results in some very simple cases that may point to more general applications, and to examine the extent to which structural parameters of graphs may provide information related to fixation probabilities and other questions of interest.

Graph parameters considered are eigenvalues of the graph Laplacian, subset conductances, graph communicability (the Estrada parameter) and measures of vertex centralities, and random walk hitting times. Choice of graphs for study has been both pragmatic and strategic: we have chosen graphs that are simple to analyse, yet exhibit behaviours suggestive for more general cases.

The graphs considered are biased cycles, dual circular flows, partial bipartite graphs and single-link coupled graphs. While cycles are perhaps the simplest graphs to consider, biased cycles display interesting properties, e.g. providing a clear case of asymmetry in the graph Laplacian that shows an analogy to rotation/angular momentum. In addition, results obtained for cycles may be useful for analysis of more complex graphs in which interaction cycles can be identified, the simplest case being dual circular flows, which can be thought of as composed of an ensemble of cycles. Dual circular flows, in turn, constitute a large family of graphs that include star graphs [4,22,25], funnel graphs [4,22,23], cascades [23] and layered networks [33]. In addition, we use them to provide an example of how the edge weights of a graph can be tuned to match specified global properties such as temperature profile. Finally, the partial bipartite and single-link graphs form the two extremes of a class of graphs with vertex set the union of two components *V*_*s*_ and *V*_*n*_ with linkages between these components through subsets *S*_*k*_⊆*V*_*s*_ and *S*_*m*_⊆*V*_*n*_ having, respectively, *k* and *m* elements. In terms of application, this family of graphs can provide models for polarized populations in which communication between different subpopulations takes place through subsets of representatives.

While illustrated here for only a small numbers of vertices, many of our results hold for graphs of arbitrary size. In particular, theorems (4.1), (4.3), (5.1) and (6.1) giving Laplacian eigenvalues for cycles, dual circular flows and partial bipartite graphs; equation 7.1, giving the characteristic polynomial for the Laplacian of a single-link graph; conjectures (6.2) and (6.3) on hitting times and communicability for partial bipartite graphs; and formulae derived for conductances are valid for any number of vertices.

Partial bipartite graphs are of particular interest. These graphs have only three non-zero Laplacian eigenvalues and which of these is smallest or largest depends on the values of the connection parameters 1−*p* and 1−*q* between the two subsets of vertices. Equality of all three eigenvalues occurs at the value of *p* and *q* for which the conductances of each graph component are equal and, although no proof is available, this appears to correspond to the condition for equality of single vertex fixation probabilities. Further, given conjecture (6.3), these parameter values give the minimum value of the communicability and are the condition for equal probability measures on paths going in either direction between graph components.

The notation used is that vertices are labelled with lower case indices ranging over the number of vertices. Thus, *v*_*k*_ labels the *k*th vertex of a graph. The single vertex fixation probability for the *k*th vertex is labelled by *x*_2^*k*−1^_ with 2^*k*−1^ the base 10 form of the binary number associated with a single one at the *k*th vertex.

## 3. Measures of graph characteristics

This section briefly describes the structural measures for graphs that are considered in this paper.

### 3.1 Eigenvalues of the Laplacian

The graph Laplacian of a graph *G* with edge weight matrix *W* is defined in Barbosa *et al.* [34] as Δ=*Φ*^1/2^(*I*−*W*)*Φ*^−1/2^, where *Φ* is a diagonal matrix with entries given by the solution of *ϕ*⋅*W*=*ϕ*. The smallest eigenvalue of Δ is always 0 and the eigen-spectrum satisfies *λ*_0_=0≤*λ*_1_≤⋯≤λ_*N*−1_≤2. The number of zero eigenvalues gives the number of disconnected components of the graph. Thus, if λ_1_ is non-zero the graph is connected and the value of λ_1_ gives a measure of the difficulty involved in cutting the graph into disconnected parts.

### 3.2 Subset conductances

For a graph (*G*, *W*) with vertex set *V* the conductance, also known as the Cheeger constant [35], is defined as

3.1 $$C_{G,W}=\underset{S\subset V,\;S\neq 0}{\min}\left(\frac{N}{\left|S||G\setminus S\right|}\sum_{\begin{array}{c} i\in S\\ j\in G\setminus S \end{array}}w_{ij}\right).$$

For a subset of vertices *S*, the conductance of *S* is

3.2 $$C_{G,W}(S)=\frac{N}{\left|S||G\setminus S\right|}\sum_{\begin{array}{c} i\in S\\ j\in G\setminus S \end{array}}w_{ij}.$$

The conductance of *S* is inversely related to the mixing time for a Markov process starting at a vertex in *S*.

### 3.3 Communicability

This concept was introduced in 2008 by Estrada & Hatano [36] in order to describe situations ‘in which a perturbation on one node of [a] network is ‘felt’ by the rest of the nodes with different intensities.’ [37], p. 92. The communicability between vertices *i* and *j* of a graph is defined as a weighted sum of all paths starting at vertex *i* and ending at vertex *j*, with weighting that favours shorter paths. This allows definition of a variety of communicability functions with the general form $P=\sum_{k=0}^{\infty }c_{k}A^{k}$, where *A* is the graph adjacency matrix and the coefficients *c*_*k*_ are chosen such that the series converges, gives greater weight to shorter paths (smaller *k* values), and yields real, non-negative values for the matrix elements of *P*. As sums over loops of all lengths starting and ending at a vertex, the diagonal elements of *P* represent a measure of vertex centrality [37].

One choice for coefficients is *c*_*k*_=1/*k*!, yielding *P*=*e*^*A*^, and the communicability is defined as Tr(e^*A*^/*N*), i.e. the average vertex centrality. Since cases considered in this paper involve probabilistically weighted edges, the matrix *A* is replaced by the edge weight matrix *W*. Hence the matrix function used is *V* =*e*^*W*^ and the *i*,*j* element of this matrix is the weighted sum of probabilities for paths of length *k* starting at *i* and ending at *j*, for 0≤k≤∞. We still refer to Tr(e^*W*^/*N*) as communicability, however. In most cases, *e*^*W*^ cannot be computed directly and the sixth-order approximation *e*^*W*^∼*I*+*W*+1/2*W*^2^+1/6*W*^3^+1/24*W*^4^+1/120*W*^5^+1/720*W*^6^ is used.

### 3.4 Expected hitting times

Following Kemeny & Snell [38], the matrix *M* of expected hitting times for a graph (*G*,*W*) is computed using the fundamental matrix of the graph, defined by *Z*=[*I*−(*W*−*A*)]^−1^ where *W* is the edge weight matrix and *A* is the matrix with columns equal to the stationary probability of a random walk on *G*. If *J* is the matrix of all ones and Z* is the diagonal matrix with $Z_{ii}^{*}=Z_{ii}$, then [38]

3.3 $$M=(I-Z+JZ^{*})D.$$

The *h*_*ij*_ element of *M* is the expected value of the random variable describing the number of iterations for a random walk that starts at vertex *i* to first reach vertex *j*.

## 4. Biased cycles

This section begins with consideration of biased cycles, that is, cycles in which there is a probabilistically preferred direction.

**Figure 1.**
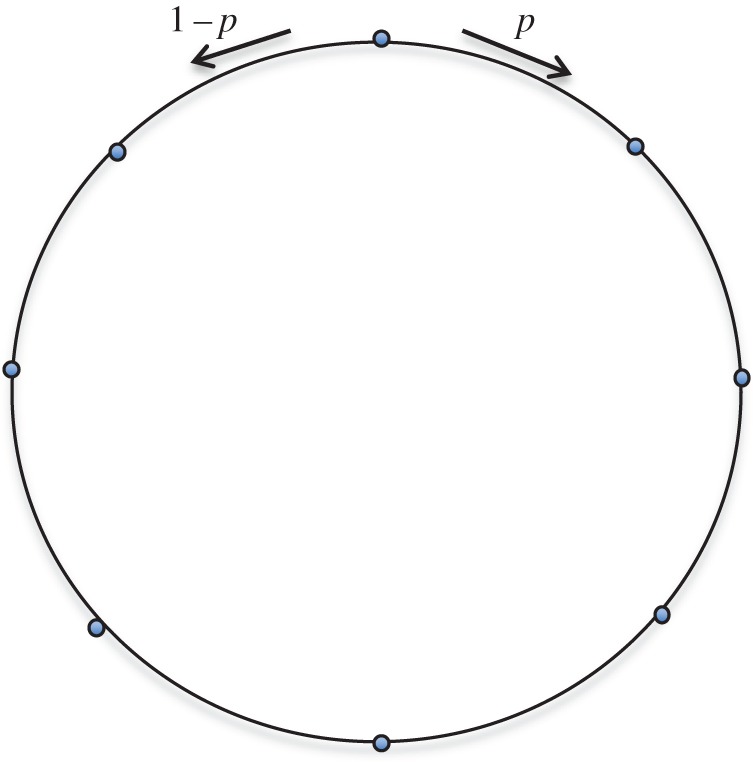
Counter-clockwise probabilities are *p* and clockwise probabilities are 1−*p*.

Figure 1 shows the general form of a biased cycle. If the parameter *p* equals one-half it becomes a balanced cycle, while *p*=1 yields a clockwise cycle and *p*=0 yields a counter-clockwise cycle. The weight matrix *W* is doubly stochastic; hence, by the Isothermal theorem, the single vertex fixation probability equals the Moran probability, although time to fixation will differ [39].

**Figure 2.**
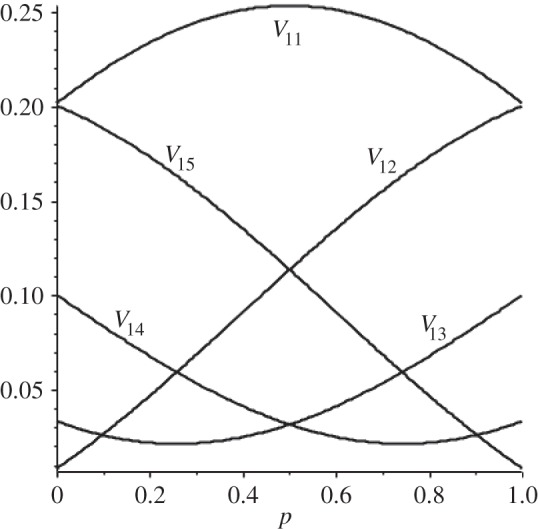
Entries in *e*^*W*^=Circ(*V*_11_,*V*_12_,*V*_13_,*V*_14_,*V*_15_).

From equation (3.2), it is easy to see that the conductance of any single connected block of *k* vertices in a length *N* biased cycle equals *N*/*k*(*N*−*k*). Also, the matrix *e*^*W*^ is circulant: *e*^*W*^=circ(*V*_11_,*V*_12_,*V*_13_,*V*_14_,*V*_15_). Since the diagonal elements of *W*^*k*^ are zero if *k* is odd and symmetric in *p* and 1−*p* if *k* is even, Tr(e^*W*^) will be symmetric in *p* and 1−*p* with maximum value at *p*=1/2. Figure 2 shows entries of *e*^*W*^/5 for *N*=5.

This figure is symmetric about *p*=1/2. *V*_*ii*_=*V*_*jj*_ for all *i*,*j*, hence Tr(e^*W*^/5)=*V*_11_ and *V*_12_=*V*_15_,*V*_13_=*V*_14_ at 1/2. The diagonal elements *V*_11_ are substantially greater than the off diagonal elements with the exceptions *V*_15_=*V*_11_ at *p*=0 and *V*_12_=*V*_11_ at *p*=1.

Since the steady state for the matrix *W* is homogeneous, the Laplacian matrix for a biased cycle is Δ=*I*−*W*. Surprisingly, the characteristic polynomial for Δ is rather complicated. For the biased cycle on *N* vertices,

4.1 $$w_{ij}=\left\{\begin{array}{ll} p & j=i+1\;\mathrm{mod}(N)\\ 1-p & j=i-1\;\mathrm{mod}(N)\\ 0 & \text{otherwise.} \end{array}\right.$$

Proof of theorems (4.1) and (4.3) is tedious but straight forward.

**Theorem 4.1**

*Let C*(*N, p*) *be a biased cycle on N vertices. The characteristic polynomial for* Δ(*C*(*N*, *p*)) *is*

4.2 $$\begin{array}{rl} & \sum_{k=0}^{(N-1)/2}{\left(-1\right)}^{k}C_{k}^{N}p^{k}{\left(1-p\right)}^{k}{\left(\lambda -1\right)}^{N-2k}+p^{N}+{\left(1-p\right)}^{N}\;N\;odd\\ & \sum_{k=0}^{(N-2)/2}{\left(-1\right)}^{k}C_{k}^{N}p^{k}{\left(1-p\right)}^{k}{\left(\lambda -1\right)}^{N-2k}-K_{N}\;\;\;N\;even\\ & K_{N}=\left\{\begin{array}{ll} {\left[p^{N/2}-{\left(1-p\right)}^{N/2}\right]}^{2} & N\equiv 0\;\mathrm{mod}(4)\\ {\left[p^{N/2}+{\left(1-p\right)}^{N/2}\right]}^{2} & N\equiv 2\;\mathrm{mod}(4), \end{array}\right. \end{array}$$

*where the coefficients satisfy* $C_{0}^{N}=1,$ $C_{1}^{N}=N$ *and* $C_{2k}^{N}=C_{2k}^{N-1}+C_{2k-1}^{N-2},C_{2k+1}^{N}=C_{2k+1}^{N-1}+C_{2k}^{N-2}.$

**Corollary 4.2**

*If p*=0 *or p*=1, *equations* (*4.2*) *reduce to* (λ−1)^*N*^=1(*N* *even*),(λ−1)^*N*^=−1(*N* *odd*) *and the eigenvalues of* Δ *are given in terms of the Nth roots of unity*: λ=1+*e*^2*πki*/*N*^ *or* λ=1−*e*^2*πki*/*N*^ *as N is even or odd, respectively*.

**Theorem 4.3**

*Let* Δ_*A*_ *be the antisymmetric part of* Δ. *For a biased cycle, all eigenvalues of* Δ_*A*_ *are either zero or are imaginary and contain a factor of* 1−2*p*.

**Figure 3.**
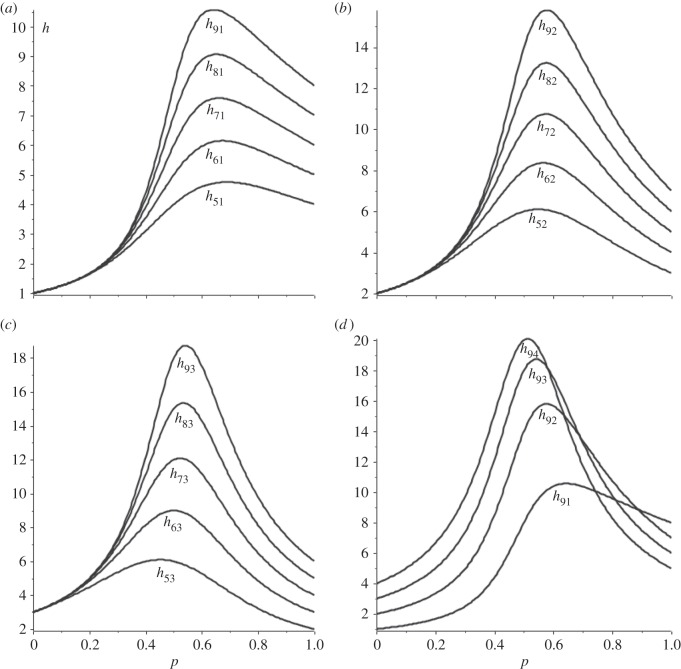
Hitting times for single vertex transition in counter-clockwise direction as function of *p* for *N*=5–9 (from bottom). (*a*) single-step, (*b*) double-step, (*c*) three-step, (*d*) *N*=9 case for one through four-step transitions (bottom to top).

If 1 – 2*p* is negative then there is a clockwise bias in the cycle, whereas if it is positive there is a counter-clockwise bias. This bias shows up clearly in the hitting times. Figure 3*a*−*c* shows hitting times for the biased cycle as a function of *p* for one-, two- and three-step transitions in the counter-clockwise direction. The shape of these curves is related to the possibility of a *k*-step transition occurring either as a counter-clockwise transition of *k* steps or a clockwise transition of *N*−*k* steps. Figure 3*d* shows the one-, two-, three- and four-step hitting times in the counter-clockwise direction for the *N*=9 biased cycle.

The value of *p* at which the peak hitting time occurs gives a measure of the bias of the cycle. For example, for *N*=5 the single-step transition in the counter-clockwise direction is favoured for p≲0.68806, whereas for *p*>0.68806 the four-step transition in the clockwise direction is favoured. Because of the symmetry between *p* and 1−*p*, the peak value of *p* starting from the symmetrically placed vertex will be one minus the peak value starting from a given vertex. Referring to figure 1, if vertex *v*_1_ is located at the top of a five cycle then the *p* value of 0.68806 characterizes vertex *v*_2_ while vertex *v*_5_ will be characterized by a peak *p*-value of 0.31194. Likewise, the two- and three-step graphs for *N*=5 are mirror images reflected about *p*=1/2.

## 5. Dual circular flows

In Voorhees [22] and Voorhees & Murray [23], a class of graphs called circular flows is defined and in Voorhees & Murray [23] a number of results concerning circular flows are given, including entropy computations and demonstration that this family of graphs contains many cases in which the single vertex fixation probability is enhanced with respect to the Moran probability only for limited values of the fitness parameter *r*≥1. In these cases, graph edges were directed in a single direction and the graphs were viewed in terms of probability flow through a series of layers. Here this is generalized to dual circular flows, that is, circular flows in which directed edges exist in both directions.

**Figure 4.**
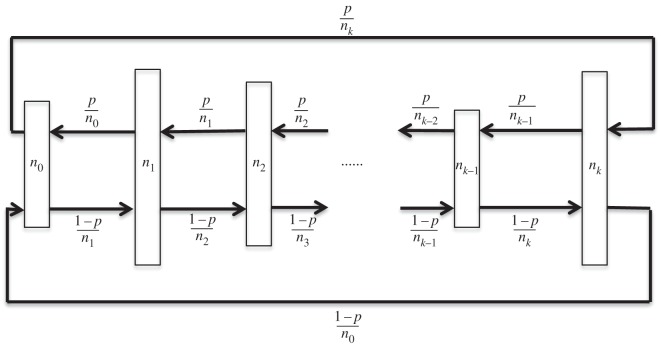
Schematic of a *k*+1 level dual circular flow. If *p*=0 or 1 this reduces to the simple homogeneous circular flow defined in Voorhees [22] and Voorhees & Murray [23].

For simplicity, only the case of uniform right- and left-directed edge weights is considered although this condition will be dropped when we consider tuning a graph. A length *k*+1 dual circular flow is illustrated schematically in figure 4.

The state space for this system is the set of vectors {(*m*_0_,*m*_1_,…,*m*_*k*_)|0≤*m*_*i*_≤*n*_*i*_}, where *m*_*i*_ indicates the number of mutants in the *i*th subset of the graph, which contains *n*_*i*_ vertices. Using the notation of Voorhees [22], *x*_*ι*(***m***)_ indicates the fixation probability of the state ***m***, whereas *x*_(***m***,*m*_*i*_±1)_ represents the fixation probability for the state arising from ***m*** in which the *i*th population of mutants has increased (+) or decreased (−) by 1. For a dual circular flow, equation (1.1) becomes

5.1 $$\begin{array}{rl} & \left\{\sum_{s=0}^{k}\left[\frac{\left(n_{s+1}-m_{s+1})p+(n_{s-1}-m_{s-1})(1-p\right)}{n_{s}}\right]+r\sum_{s=0}^{k}\left(n_{s}-m_{s}\right)\left[\frac{m_{s+1}p+m_{s-1}(1-p)}{n_{s}}\right]\right\}x_{\iota (\text{m})}\\ & \;-\sum_{s=0}^{k}\left[\frac{\left(n_{s+1}-m_{s+1})p+(n_{s-1}-m_{s-1})(1-p\right)}{n_{s}}\right]x_{\iota (\text{m},m_{s-1})}\\ & \;-r\sum_{s=0}^{k}\left(n_{s}-m_{s}\right)\left[\frac{m_{s+1}p+m_{s-1}(1-p)}{n_{s}}\right]x_{\iota (\text{m},m_{s+1})}=0. \end{array}$$

(All sums taken mod(*k*+1).)

**Figure 5.**
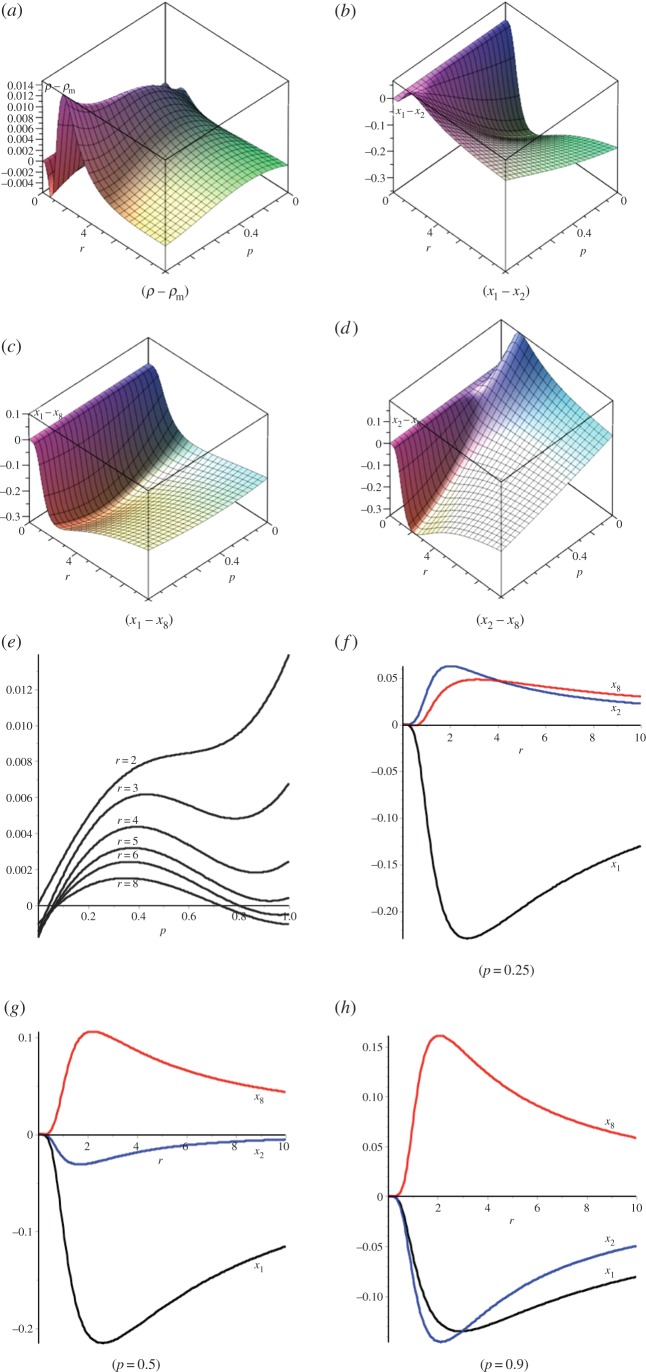
Fixation probabilities for (1,2,3) dual circular flow. (*a*) Single vertex fixation probability, (*b*) fixation probabilities *x*_1_ minus *x*_2_, (*c*) fixation probabilities *x*_1_ minus *x*_8_, (*d*) fixation probabilities *x*_2_ minus *x*_8_, (*e*) single vertex fixation probability as a function of *p* for specified *r* values and (*f*–*h*) fixation probabilities *x*_1_, *x*_2_ and *x*_8_ for specified *p*-values.

Figure 5 shows single vertex fixation probabilities for the (*n*_0_,*n*_1_,*n*_2_)=(1,2,3) dual circular flow. The *p*=0 case corresponds to the (1, 2, 3) cascade graph and the *p*=1 case corresponds to the (1, 2, 3) funnel graph. The fixation probability for the single vertex at level 0 is *x*_1_, for each of the two vertices at level 1 it is *x*_2_, and for each of the three vertices at level 2 it is *x*_8_. The overall single vertex fixation probability is *ρ*=(*x*_1_+2*x*_2_+3*x*_3_)/6 and the Moran probability is denoted *ρ*_m_.

Figure 5*a* shows the transition between fixation probabilities for cascade and funnel flows as *p* varies from 0 to 1. Figure 5*b*–*d* shows the differences between single vertex fixation probabilities from different levels (0, 1, 2). Figure 5*e* shows cross sections of figure 5*a* for specified values of *r*, whereas figure 5*f*–*h* shows plots of *x*_1_−*ρ*_m_, *x*_2_−*ρ*_m_ and *x*_8_−*ρ*_m_, respectively, as functions of *r* for specified *p*-values.

Examination of these figures indicates that starting at the single, level zero vertex always suppresses selection, starting at one of the three level-two vertices always enhances selection, relative to the Moran value, while starting at one of the two level-one vertices will enhance or suppress selection depending on the values of *p* and *r* (for example, for *p*=0.4 selection is enhanced for *r*>1). At *p*=0.25, *x*_2_>*x*_8_ for *r*<4.0471 while for *r* values greater than this *x*_8_>*x*_2_. Likewise, at *p*=0.9, *x*_2_<*x*_1_ for *r*<2.9803 and the inequality is reversed for *r* greater than this value. Thus, the two vertices at level 1 exhibit the least stability in terms of fixation behaviour.

This example illustrates the complex behaviour that appears even in simple dual circular flow graphs. In Voorhees & Murray [23], a number of cases are considered showing that the single vertex fixation probability for such graphs can display both enhancement and suppression of selection, depending on the particular value of the fitness *r*. For example, in the (1, 2, 3)-funnel graph (*p*=1), selection is only enhanced for 1<*r*<5.3695, whereas for the (1, 2, 3)-cascade (*p*=0), it is only enhanced for 1<*r*<2.0304.

The following theorem relates the characteristic polynomials for the Laplacian of all dual circular flow graphs to that of a biased cycle of the same length.

**Theorem 5.1**

*Let* (*n*_0_, *n*_1_,…,*n*_*k*_) *represent a dual circular flow with* $N=\sum_{i=0}^{k}n_{i}$. *Then the characteristic polynomials for* Δ *and* Δ_*A*_ *are just* (λ−1)^*N*−*k*−1^ *multiplying the corresponding characteristic polynomials of a length k biased cycle with bias p.*

Outline of proof

Beginning with the flow (1, 1, 2), the claim is demonstrated for this case. This is extended by induction to the flows (1, 1, *n*) and (1, …,1, *n*). With this established a further induction demonstrates the result for flows (1,…,1,*n*_*k*−1_,*n*_*k*_). Proceeding in this way, an eventual proof is obtained.

One point to note is that all eigenvalues of Δ_*A*_ again involve a factor of 1−2*p*, providing a measure of directional bias in the circular flow.

**Figure 6.**
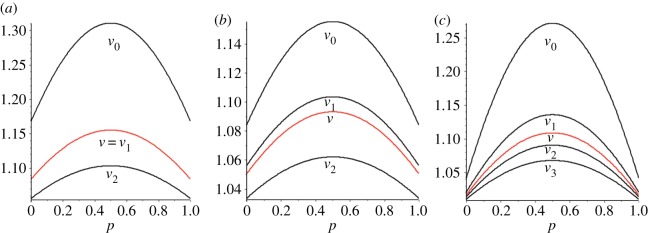
Communicability (*V* , in red) and associated vertex centralities (black) for dual circular flows: (*a*) (1,2,3) flow (note the communicability equals the *v*_1_ vertex centrality), (*b*) (2,3,5) flow and (*c*) (1,2,3,4) flow.

By equation (3.2) and inspection of figure 4, it is obvious that the conductance of level *s* in a dual circular flow equals *N*/(*N*−*n*_*s*_). Computation of the communicability and the associated vertex centralities yield results like those indicated in figure 6 which shows these plotted as functions of *p* for the dual flows 123, 235 and 1234, with *V*_*i*_ indicating the centrality of a vertex at level *i*.

Inspection of this figure indicates that the centrality of a vertex in a given layer is inversely related to the number of vertices in that layer—multiplying vertex centrality by the number of vertices in a layer shows that the centrality of a layer is proportional to the number of vertices—layers with more vertices have greater centrality. This can be understood in terms of the topological structure of these graphs—there are no edges connecting vertices within a layer. This also implies that the hitting times between layers will be the same as for a biased cycle of the same length, although hitting times between specific vertices will be substantially longer.

**Figure 7.**
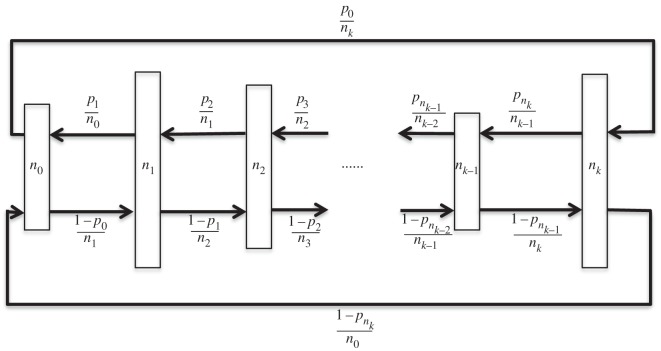
Schematic of a general *k*+1 level dual circular flow.

Dual circular flows also provide an example of the way that edge coefficients in a graph can be tuned to match desired global features. To address this, the graph of figure 4 is generalized, allowing different weight coefficients between levels. This is indicated in figure 7. The target parameter chosen for tuning is vertex temperature [4] at each level of the graph and the inter-level edge weights *p*_*i*_ will be selected to produce specified temperature profiles. In particular, conditions will be determined for which the single vertex fixation probability matches that of a complete graph.

Referring to figure 7, the temperature of a vertex at level *i* is

5.2 $$t_{i}=\frac{n_{i+1}p_{i+1}+n_{i-1}(1-p_{i-1})}{n_{i}}.$$

Hence, if a temperature profile ***t***=(*t*_0_,…,*t*_*k*_) is to be matched the following set of equations must be satisfied:

5.3 $$n_{i+1}p_{i+1}-n_{i-1}p_{i-1}=t_{i}n_{i}-n_{i-1}.$$

Two examples will illustrate the factors involved in doing this.

**Example 5.2**

A three-level flow (*n*_0_,*n*_1_,*n*_2_) with *t*_*i*_=1 for all i.

In this case, the goal is to adjust the connection coefficients *p*_*i*_ so as to produce a graph with single vertex fixation probability equal to the Moran probability. Equation (5.3) becomes

5.4 $$\left.\begin{array}{r} n_{1}p_{1}-n_{2}p_{2}=n_{0}-n_{2}\\ n_{2}p_{2}-n_{0}p_{0}=n_{1}-n_{0}\\ n_{0}p_{0}-n_{1}p_{1}=n_{2}-n_{1}. \end{array}\right\}$$

Setting *p*_2_=*p* and solving for *p*_0_ and *p*_1_ yields

5.5 $$p_{0}=\frac{n_{0}-n_{1}+n_{2}p}{n_{0}}\;\text{and}\;p_{1}=\frac{n_{0}-n_{2}+n_{2}p}{n_{1}}.$$

Since *p*_0_ and *p*_1_ are probabilities they must lie in the range [0, 1], and this requires

5.6 $$\frac{n_{1}-n_{0}}{n_{2}}\leq p\leq \frac{n_{1}}{n_{0}}\;\text{and}\;\frac{n_{2}-n_{0}}{n_{2}}\leq p\leq \frac{n_{1}}{n_{2}}.$$

For example, if (*n*_0_,*n*_1_,*n*_2_)=(1,2,3), equation (5.6) requires 1/3≤*p*≤2 and 2/3≤*p*≤2/3. From the second of these, the only value allowed for *p*=*p*_2_ is 2/3 and this yields *p*_0_=1, *p*_1_=0. Thus, the (1,2,3) flow with edge weights (*p*_0_,*p*_1_,*p*_2_)=(1,0,2/3) has the Moran single vertex fixation probability. If (*n*_0_,*n*_1_,*n*_2_)=(2,3,4), on the other hand, there is a larger range of possible edge weights: the conditions that must be satisfied are 1/2≤*p*_2_≤3/4 with *p*_0_=2*p*_2_−1/2 and *p*_1_=2(2*p*_2_−1).

If the number of levels in the graph is even (i.e. *k* is odd), an additional constraint appears. Equation (5.3) separate into two sets of equations with the left sides of each set summing separately to zero. This yields the conditions

5.7 $$\sum_{i=0}^{(k-1)/2}t_{2i}n_{2i}=\sum_{i=0}^{(k-1)/2}n_{2i+1}\;\text{and}\;\sum_{i=0}^{(k-1)/2}t_{2i+1}n_{2i+1}=\sum_{i=0}^{(k-1)/2}n_{2i}.$$

For example, if *k*=3 and *t*_*i*_=1 for all *i*, this requires *n*_0_+*n*_2_=*n*_1_+*n*_3_. In addition, each set of equations is solved in terms of a single parameter so there are two parameters in the solution. For example, in the general *k*=5 case, the equations are

5.8a $$\left.\begin{array}{r} n_{2}p_{2}-n_{0}p_{0}=t_{1}n_{1}-n_{0}\\ n_{4}p_{4}-n_{2}p_{2}=t_{3}n_{3}-n_{2}\\ n_{0}p_{0}-n_{4}p_{4}=t_{5}n_{5}-n_{4} \end{array}\right\}$$

and

5.8b $$\left.\begin{array}{r} n_{1}p_{1}-n_{5}p_{5}=t_{0}n_{0}-n_{5}\\ n_{3}p_{3}-n_{1}p_{1}=t_{2}n_{2}-n_{1}\\ n_{5}p_{5}-n_{3}p_{3}=t_{4}n_{4}-n_{3}, \end{array}\right\}$$

with constraints

5.8c $$\left.\begin{array}{r} n_{0}+n_{2}+n_{4}=t_{1}n_{1}+t_{3}n_{3}+t_{5}n_{5}\\ n_{1}+n_{3}+n_{5}=t_{0}n_{0}+t_{2}n_{2}+t_{4}n_{4}. \end{array}\right\}$$

Equations (5.8*a*,*b*) have solutions in terms of *p*_4_ and *p*_5_, respectively:

5.9 $$\left.\begin{array}{r} p_{0}=\frac{t_{5}n_{5}-n_{4}(1-p_{4})}{n_{0}},\;p_{2}=\frac{n_{4}p_{4}+n_{2}-t_{3}n_{3}}{n_{2}}\\ p_{1}=\frac{t_{0}n_{0}-n_{5}(1-p_{5})}{n_{1}},\;p_{3}=\frac{n_{5}p_{5}+n_{3}-t_{4}n_{4}}{n_{3}}. \end{array}\right\}$$

For a solution to be valid, all transition probabilities must be in [0,1] imposing the additional constraints

5.10 $$\left.\begin{array}{r} 1-\frac{t_{5}n_{5}}{n_{4}}\leq p_{4}\leq 1+\frac{n_{0}-t_{5}n_{5}}{n_{4}},\;\frac{t_{3}n_{3}-n_{2}}{n_{4}}\leq p_{4}\leq \frac{t_{3}n_{3}}{n_{4}}\\ 1-\frac{t_{0}n_{0}}{n_{5}}\leq p_{5}\leq 1+\frac{n_{1}-t_{0}n_{0}}{n_{5}},\;\frac{t_{4}n_{4}-n_{3}}{n_{5}}\leq p_{5}\leq \frac{t_{4}n_{4}}{n_{5}}, \end{array}\right\}$$

while the values of *p*_4_, *p*_5_ are also constrained to lie in [0, 1].

If edge weights exist satisfying the temperature profile *t*_*i*_=1, 0≤*i*≤*k*, then the graph with these edge weights will have the Moran fixation probability. The next example, however, demonstrates that matching a general temperature profile does not always lead to matching fixation probabilities.

**Example 5.3**

**Figure 8.**
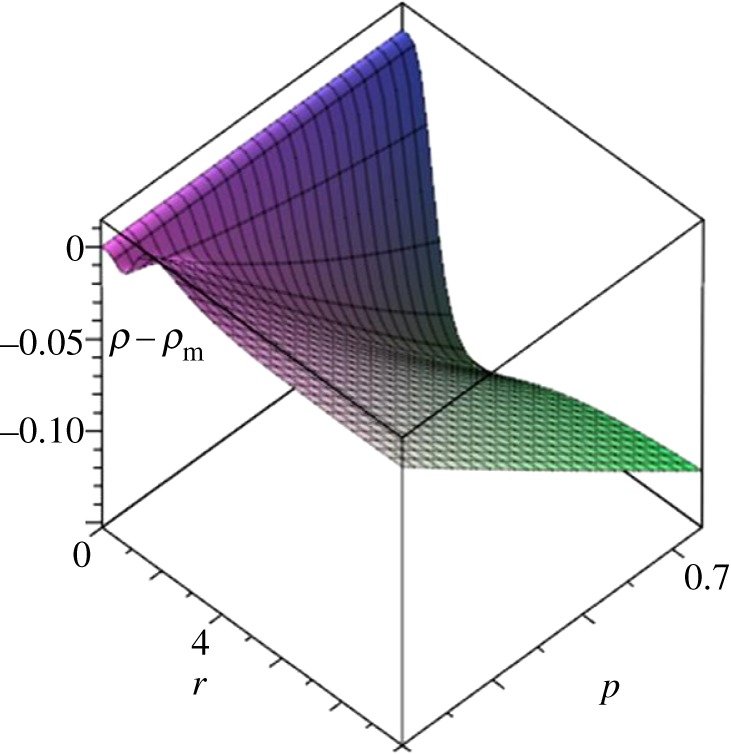
Fixation probabilities for (1,2,3) flow matching temperature profile (2,3/2,1/3).

Consider the circular flow (*n*_0_,*n*_1_,*n*_2_) with temperature profile (2, 3/2, 1/3). Any edge weights satisfying *p*_0_=3*p*_2_−2, *p*_1_=(3*p*_2_− 1)/2 will match this profile. If *p*_2_=1 then *p*_0_=*p*_1_=1 as well—this case is considered in Voorhees & Murray [23]. But all values of *p*_2_ in the interval [2/3, 1] yield valid solutions matching the given profile. Figure 8 shows a plot of the single vertex fixation probability minus the Moran probability for this range of *p*, illustrating that very different fixation probabilities result.

## 6. Partial bipartite graphs

The vertex set for a complete bipartite graph *K*_*s*,*n*_ consists of two subsets *V*_*s*_ and *V*_*n*_ containing *s* and *n* vertices, respectively, with edge set $\left\{(v_{i},v_{j})\left|v_{i}\in V_{s},\right.v_{j}\in V_{n}\right\}$. That is, every vertex in *V*_*s*_ has a link to every vertex in *V*_*n*_ and vice versa but no pairs of edges in *V*_*s*_ are linked nor are any pairs of edges in *V*_*n*_. Furthermore, if edges are directed and weighted, then all weights for edges from *V*_*s*_ to *V*_*n*_ are 1/*n* and all weights for edges from *V*_*n*_ to *V*_*s*_ are 1/*s*. This family of graphs includes the star graphs (*s*=1). In Voorhees & Murray [23], the birth–death single vertex fixation probability for the complete bipartite graph *K*_*s*,*n*_ is given as

6.1 $$\begin{array}{r} \begin{array}{rl} \rho_{K_{s,n}}(s,n) & =\left(\frac{r^{n+s-1}}{sr+n}\right)\left[\frac{(snr+n^{2}-sn+s^{2}){\left(nr+s\right)}^{n-s-1}}{P(s,n)}\right]\\ P(s,n) & =\frac{r^{n+s}{\left(nr+s\right)}^{n-s}-{\left(sr+n\right)}^{n-s}}{r^{2}-1}. \end{array} \end{array}$$

**Figure 9.**
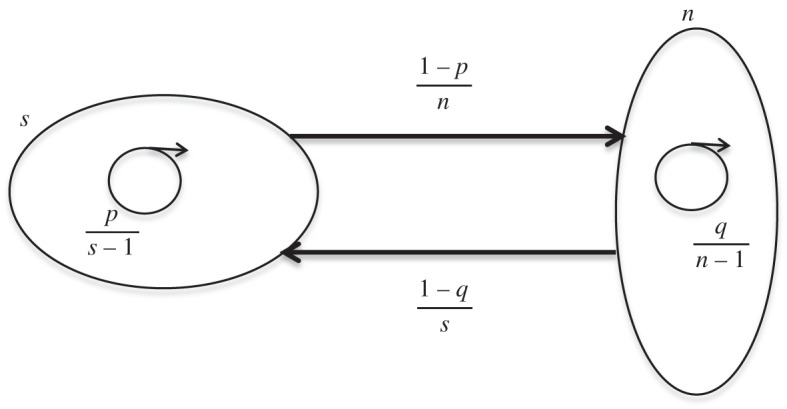
Schematic of a partial bipartite graph.

Here we consider the more general case of partial bipartite graphs, as illustrated in figure 9. This figure shows two subsets *V*_*s*_ and *V*_*n*_ of vertices with |*V*_*s*_|=*s* and |*V*_*n*_|=*n*. Within each subset, every vertex connects equally to every other vertex with probabilities *p*/(*s*−1) and *q*/(*n*−1), respectively. In addition, every vertex in *V*_*s*_ connects to every vertex of *V*_*n*_ with the probability (1−*p*)/*n* and every vertex of *V*_*n*_ connects to every vertex of *V*_*s*_ with probability (1−*q*)/*s*.

The edge weight matrix for this graph has block form:

6.2 $$W=\left(\begin{array}{cc} \frac{p}{s-1}(J_{s,s}-I_{s}) & \frac{1-p}{n}J_{s,n}\\ \frac{1-q}{s}J_{n,s} & \frac{q}{n-1}(J_{n.n}-I_{n}) \end{array}\right),$$

where *J*_*i*,*j*_ is the *i* by *j* matrix of all ones and *I*_*j*_ is the *j* by *j* identity matrix. The normalized steady-state solution for ***v***⋅*W*=***v*** is

6.3 $$\text{v}=\frac{1}{sn(2-p-q)}(n(1-q),\ldots ,n(1-q),s(1-p),\ldots ,s(1-p)),$$

with *s* entries of *n*(1−*q*) and *n* entries of *s*(1−*p*). The Laplacian matrix and its antisymmetric part have block form

6.4 $$\begin{array}{rl} \Delta & =I_{N}-\left(\begin{array}{cc} I_{s}-\frac{p}{s-1}(J_{s,s}-I_{s}) & \frac{\sqrt{\left(1-p)(1-q\right)}}{n}J_{s,n}\\ \frac{\sqrt{\left(1-p)(1-q\right)}}{s}J_{n,s} & I_{n}-\frac{q}{n-1}(J_{n.n}-I_{n}) \end{array}\right)\\ \Delta_{A} & =\frac{(n-s)\sqrt{\left(1-p)(1-q\right)}}{sn}\left(\begin{array}{cc} 0 & J_{s,n}\\ J_{n,s} & 0 \end{array}\right). \end{array}$$

Proof of the next theorem follows directly from the form of these matrices.

**Theorem 6.1**

*The characteristic polynomials for* Δ *and* Δ_*A*_ *of the N vertex partial bipartite graph with edge weight matrix W given in equation* (*6.2*) *are*

6.5 $$\left.\begin{array}{r} \lambda {\left(\lambda -\frac{s-1+p}{s-1}\right)}^{s-1}{\left(\lambda -\frac{n-1+q}{n-1}\right)}^{n-1}(\lambda -2+p+q)\\ \lambda^{N-2}\left[\lambda^{2}+\frac{{\left(n-s\right)}^{2}}{sn}(1-p)(1-q)\right]. \end{array}\right\}$$

Thus, Δ has eigenvalues of 0, (*s*−1+*p*)/(*s*−1) with multiplicity *s*−1, (*n*−1+*q*)/(*n*−1) with multiplicity *n*−1, and 2−*p*−*q*, whereas Δ_*A*_ has *N*−2 zero eigenvalues with the remaining two eigenvalues imaginary and equal to $\pm \text{i}\left|n-s\right|\sqrt{(1-p)(1-q)/sn}$.

The total probability flow from *V*_*s*_ to *V*_*n*_ equals *s*(1−*p*) while the total flow from *V*_*n*_ to *V*_*s*_ is *n*(1−*q*). In the bipartite case, the subsets of interest are *V*_*s*_ and *V*_*n*_. The right and left conductance of the graph are thus defined as

6.6 $$\left.\begin{array}{r} C(V_{s})=\left(\frac{s+n}{sn}\right)\sum_{i\in V_{s},j\in V_{n}}w_{ij}=\frac{N}{n}(1-p)\\ \;\text{and}\;C(V_{n})=\left(\frac{s+n}{sn}\right)\sum_{i\in V_{n},j\in V_{s}}w_{ij}=\frac{N}{s}(1-q). \end{array}\right\}$$

The conductance of a set of vertices *S* is inversely related to the mixing time of a Markov process starting from a vertex in *S*. Thus, *C*(*V*_*s*_)≥*C*(*V*_*n*_) implies that the mixing time starting from a vertex in *V*_*s*_ will be less than that starting from a vertex in *V*_*n*_. The condition for *C*(*V*_*s*_)≥*C*(*V*_*n*_) is

6.7 $$1-p\geq \frac{n}{s}(1-q).$$

**Figure 10.**
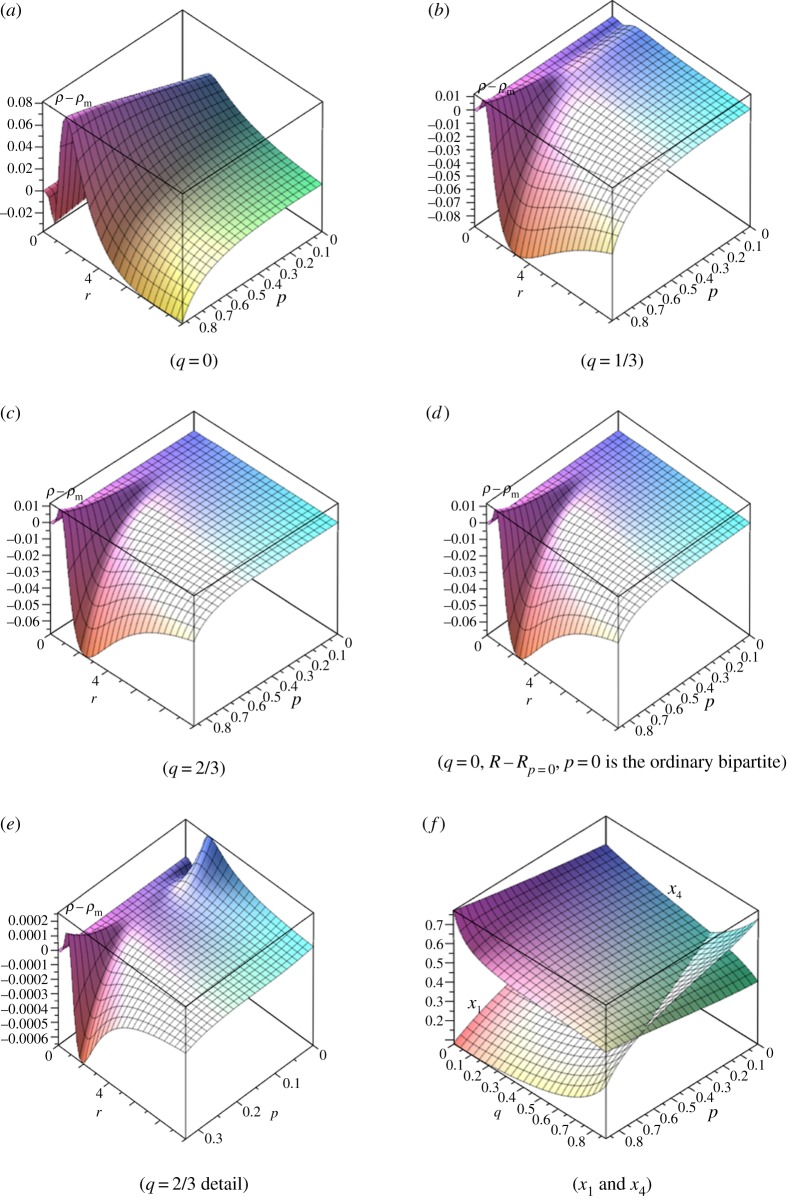
Fixation probabilities minus Moran probabilities for (2, 5) partial bipartite graph for varying *q* values (*a*–*d*), (*e*) detail of (*c*) (0≤*p*≤1/3) and (*f*) the *x*_1_ (vertex in *V*_2_ component) and *x*_4_ (vertex in *V*_5_ component) probabilities for *r*=2.

Figure 10 shows the single vertex probability for the (2, 5) partial bipartite graph for fixed values of *q* with *p* and *r* as variables and for *r*=2 with *p* and *q* as variables.

**Figure 11.**
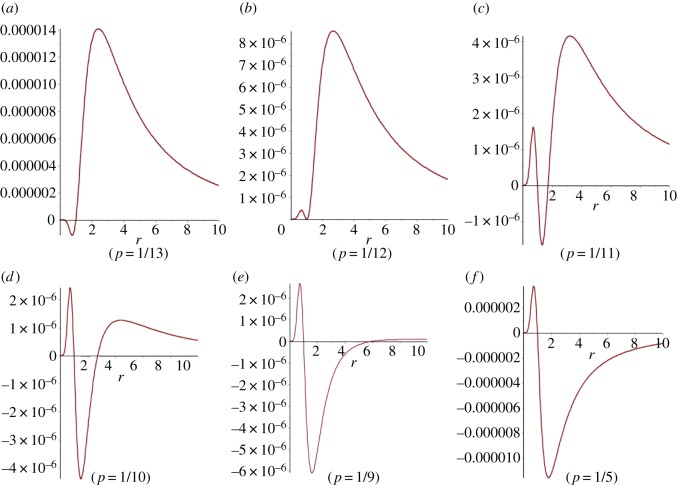
Cross sections of figure 10*e*.

Although figure 10*c* and 10*d* appear quite similar there is a significant difference. In figure 10*d* the *p*=0 line is at 0, whereas in figure 10*c* this is not quite the case, as indicated in the detail of figure 10*e*. Figure 11 shows cross sections of figure 10*e*. While the difference from the Moran probability is extremely small, these figures show an unusual effect in which selection is enhanced for fitness values less than one, suppressed for a limited range of values greater than one and then enhanced again. The graphs of figure 11 illustrate a transition between three distinct regimes of behaviour. In the first, selection is suppressed for 0<*r*<1 and enhanced for *r*>1 (pattern A, table 1). In the second, selection is enhanced for 0<*r*<1, and there is a value $r_{\max}$ dependent on *p*, such that selection is suppressed for fitness in the range [1, $r_{\max}$] and enhanced for fitness greater than $r_{\max}$ (pattern C, table 1). In the third, selection is enhanced for 0<*r*<1 and suppressed for *r*>1 (pattern D, table 1). When other values of *q* are considered, similar sorts of transitions appear, with a fourth region in which selection is suppressed for 0<*r*<1, enhanced for fitness in the range [1, $r_{\max}$] and suppressed for $r>r_{\max}$ (pattern B, table 1). Table 1 shows comparative *q*- and *p*-values illustrating this effect, which will be discussed further in §7.

**Table 1.** Patterns of single vertex fixation probability in (2, 5) partial bipartite graph.

*q*	pattern A	pattern B (*p*,*r*_max_)	pattern C (*p*,*r*_max_)	pattern D
0	*p*<0.5	(0.5,257.0019), (0.55,23.5695), (0.6,12.5060), (0.75,5.3575), (0.875,3.7144), (1,2.8845)		
0.125	*p*<0.445	(0.445,261.1673), (0.45,91.6745), (0.55,6.2814), (0.6,4.1189), (0.75,1.3160), (0.85,1.0000)		*p*>0.85
0.3333	*p*<0.3375	(0.3375,109.1445), (0.3402,28.3141), (0.35,11.2907), (0.3667,4.9994), (0.4,2.1249), (0.5,1.0000)		*p*>0.5
0.5	*p*<0.2		(0.2075,1.0260), (0.208,1.1176), (0.2085,1.2023), (0.21,1.4381), (0.215,2.2926), (0.228,404.7369)	*p*>0.228
0.625	*p*<1/16			*p*>1/16
0.6667	*p*<0.0833		(0.0833,1.0000), (0.0999,1.7239), (0.1,2.7687), (0.1111,5.9870), (0.117,247.4142) (0.118,42.2788,175.4007)	*p*>0.125
0.875	*p*>0.585		(0.48,821.1031), (0.49,13.0399), (0.5,7.0984), (0.51,5.0663), (0.55,2.3417), (0.585,1.2061)	*p*<0.48

The *q*=0.6667, *p*=0.118 case is an anomaly in that selection is enhanced for the two regions 0<*r*<1 and 42.2788<*r*<175.4007 and suppressed for larger *r* (the enhancement is of the order of 10^−11^ in the second region).

**Figure 12.**
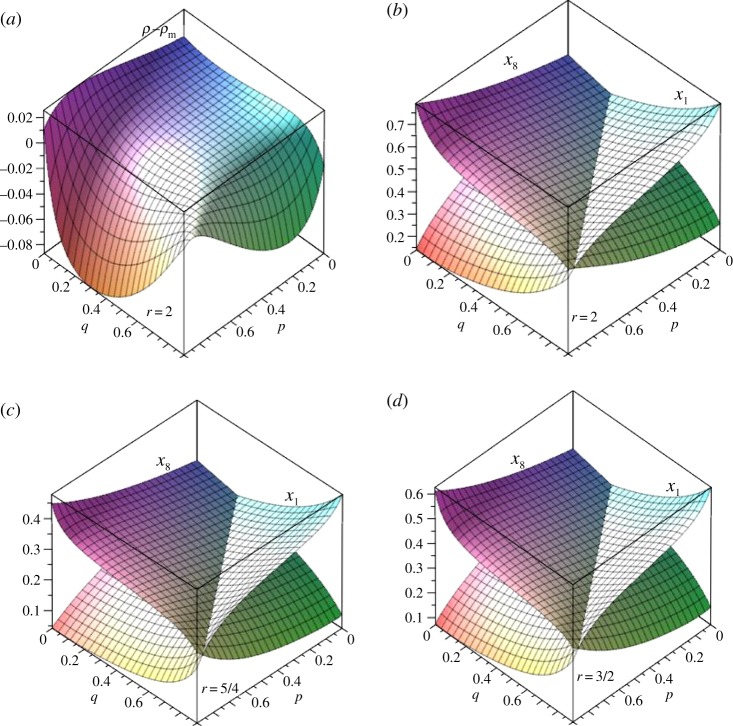
Single vertex fixation probabilities for (3,4) partial bipartite graph with *r*=5/4, 3/2 and 2. (*a*) Fixation probability minus Moran probability with *r*=2, (*b*–*d*) fixation probabilities starting on three sides (sheet that is lower at *q*=0) compared to that starting on fourth side for *r*=5/4, 3/2, 2.

Figure 12 shows probabilities for the (3,4) partial bipartite graph as functions of *p* and *q* for *r* taking values 5/4, 3/2 and 2. In all cases, the ranges of *p* and *q* are [0, 7/8] in order to avoid a singular that arises if *p* or *q* is 1.

Figure 12*b*–*d* plots *x*_1_ and *x*_8_, the single vertex probabilities for a vertex starting in the three vertex subset and the four vertex subset, respectively. The equation for *x*_1_−*x*_8_=0 contains over 350 terms in products of *p* and *q* with coefficients of up to 20 digits. Nevertheless, a simple solution exists and is the same for all three values of *r* considered: *q*=(1+3*p*)/4. This compares exactly with the condition based on conductances given in equation (6.7). Likewise, for the (2, 5) case the *x*_1_−*x*_4_=0 line is *q*=(3+2*p*)/5, which again is the same condition as given by equation (6.7). This suggests that equation (6.7) provides a sharp differentiation between fixation probabilities for vertices starting in one or the other of the two graph components *V*_*s*_ and *V*_*n*_.

If the condition of equation (6.7) is satisfied the subset *V*_*s*_ will be said to be evangelical relative to *V*_*n*_ while if this condition is not satisfied *V*_*s*_ is isolated relative to *V*_*n*_. In the extreme case *p*=1, we will say that *V*_*s*_ is quarantined.

Estimates of hitting times were computed for the (*s*,*n*) partial bipartite graph for a number of examples and the form of these cases suggests the following conjecture.

**Conjecture 6.2**

*Let G be an* (*s, n*) *partial bipartite graph with h*(*s, s*) *and h*(*n, n*) *the respective hitting times between pairs of vertices in V*_*s*_ *and V*_*n*_, *and h*(*s, n*) *the hitting time starting from a vertex in V*_*s*_ *and ending at one in V*_*n*_ *while h*(*n, s*) *is the hitting time starting from a vertex in V*_*n*_ *and ending at one in V*_*s*_. *Then*

6.8 $$\left.\begin{array}{r} h(s,s)=\frac{s(s-1)(2-p-q)}{\left(1-q)(s-1+p\right)},\;h(n,n)=\frac{n(n-1)(2-p-q)}{\left(1-p)(n-1+q\right)},\\ h(s,n)=\frac{(n-1)(2n-1)-{\left(n-1\right)}^{2}p-n(n-2)q}{\left(1-p)(s-1+q\right)}\\ \;\text{and}\;h(n,s)=\frac{(s-1)(2s-1)-{\left(s-1\right)}^{2}q-s(s-2)p}{\left(1-q)(n-1+p\right)}. \end{array}\right\}$$

Given this conjecture, the condition for *h*(*s*,*s*)≥*h*(*n*,*n*) is

6.9 $$q\geq \frac{(s-1)(n-1)(n-s)+(n-1)[n+s(s-1)]p}{sn(n-1)-[n(n-1)-s(s-1)](1-p)}.$$

If (*s*,*n*)=(3,4) then equation (6.9) becomes *q*≥(1+*p*)/(5+*p*).

Note that the hitting times *h*(*s*,*s*) and *h*(*n*,*n*) both involve a factor of 2−*p*−*q* which is an eigenvalue of the Laplacian. If *p* and *q* are sufficiently small, 2−*p*−*q* is the largest eigenvalue. From equation (6.5), ‘sufficiently small’ requires

**Figure 13.**
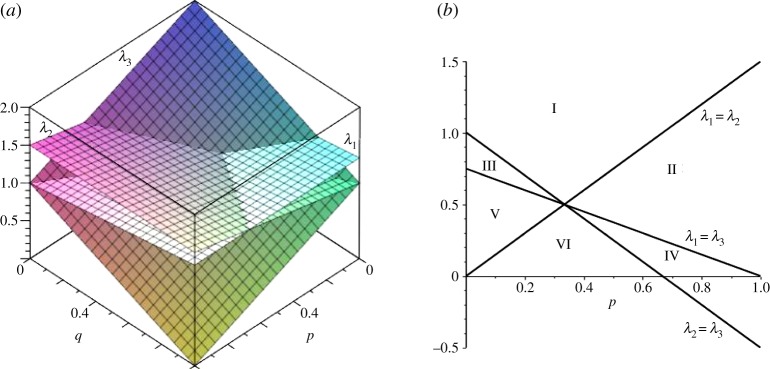
(*a*) Eigenvalues of Δ for (3,4) partial bipartite graph as functions of (*p*,*q*). λ_1_=(3+*q*)/3,λ_2_=(2+*p*)/2,λ_3_=2−*p*−*q*. (*b*) Equal value lines for eigenvalues of Δ for (3,4) partial bipartite graph.

6.10 $$q\leq \frac{(1-p)s-1}{s-1}\;\text{and}\;p\leq \frac{(1-q)n-1}{n-1},$$

with equality yielding *p*=(*s*−1)/(*N*−1), *q*=(*n*−1)/(*N*−1). More generally, eigenvalues of the Laplacian can be plotted as functions of *p* and *q*, as illustrated in figure 13*a* for (*s*,*n*)=(3,4), and the curves for equality of pairs of the non-zero eigenvalues can be computed, showing *q* as a function of *p* as in figure 13*b*.

The point of equality for all three eigenvalues is (*p*,*q*)=(1/3,1/2).

Ranking eigenvalues according to size for the six different regions in this graph is shown in table 2.

**Table 2.** Size order of eigenvalues of Δ for regions defined in figure 13*b*.

region in figure 13	non-zero eigenvalue order
I	λ_3_, λ_1_, λ_2_
II	λ_3_, λ_2_, λ_1_
III	λ_1_, λ_3_, λ_2_
IV	λ_2_, λ_3_, λ_1_
V	λ_1_, λ_2_, λ_3_
VI	λ_2_, λ_1_, λ_3_

In the limiting case of *p*=*q*=1, the eigenvalue 2−*p*−*q* becomes zero and the graph separates into two disconnected components. Care is required, however, to determine how *h*(*s*,*s*) and *h*(*n*,*n*) behave in this limit since the factors (2−*p*−*q*)/(1−*p*) and (2−*p*−*q*)/(1−*q*) give results that depend on how the limit (*p*,*q*)→(1,1) is approached. For example, suppose that *p*=*q*^*k*^. Then 2−*p*−*q*=2−*q*−*q*^*k*^=(1−*q*)(2+*q*+*q*^2^+⋯+*q*^*k*−1^) and at *q*=1*h*(*s*,*s*)=(1+*k*)(*s*−1) and likewise *h*(*n*,*n*)=(1+*k*)(*n*−1). These differ from the hitting times for size *s* and size *n* complete graphs, which are *s*−1 and *n*−1, respectively. The way to deal with this in computing *h*(*s*,*s*) is to set *p*=1 before going to the limit (or, in computing *h*(*n*,*n*) setting *q*=1). Otherwise there is always the possibility of transitions between *V*_*s*_ and *V*_*n*_ giving additional contributions to hitting times.

Exact computation of *e*^*W*^ is possible for partial bipartite graphs and examination of the cases (2, 3), (3, 4), (2, 5), (3, 5) and (4, 5) suggests the following.

**Conjecture 6.3**

*Let G be an* (*s, n*) *partial bipartite graph, V*_*ss*_ *and V*_*nn*_ *the diagonal elements of e*^*W*^ *corresponding to vertices in V*_*s*_ *and V*_*n*_, *with V*_*sn*_ *and V*_*ns*_ *refer to off block diagonal elements. Then*

6.11 $$\left.\begin{array}{r} V_{ss}=\frac{se}{2-p-q}\left[1-q+(1-p)e^{p+q-2}\right],\;V_{sn}=\frac{s(1-p)e}{2-p-q}\left[1-e^{p+q-2}\right]\\ V_{ns}=\frac{n(1-q)e}{2-p-q}\left[1-e^{p+q-2}\right]\;\text{and}\;V_{nn}=\frac{ne}{2-p-q}\left[1-p+(1-q)e^{p+q-2}\right]. \end{array}\right\}$$

In addition,

6.12 $$\text{Tr}(e^{W})=e\left[1+e^{p+q-2}+(n-1)e^{-\left(n-1+q\right)/\left(n-1\right)}+(s-1)e^{-\left(s-1+p\right)/\left(s-1\right)}\right].$$

Setting partial derivatives of Tr(e^*W*^) with respect to *p* and *q* to zero and solving for *q* yields

6.13 $$q=\frac{s-1-sp}{s-1}\;\text{and}\;q=\frac{\left(n-1)(1-p\right)}{n}.$$

Setting these equal indicates that the minimum value of Tr(e^*W*^) occurs at

6.14 $$(p,q)=\left(\frac{1}{n+s-1}\right)(s-1,n-1),$$

which is the condition for eigenvalue equality in equation (6.10).

**Figure 14.**
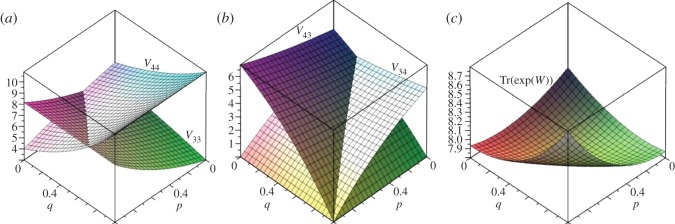
Plots of (*a*) *V*_33_ and *V*_44_, (*b*) *V*_34_ and *V*_43_, and (*c*) Tr(*e*^*W*^) for (3,4) partial bipartite graph.

Figure 14 shows plots of *V*_*ss*_ and *V*_*nn*_, *V*_*sn*_ and *V*_*ns*_, and Tr(e^*W*^) for (*s*,*n*)=(3,4).

In figure 14*a*, *V*_33_ is lowest at *p*=0, *q*=1 and in figure 14*b*, *V*_*sn*_ is zero at *p*=1, whereas *V*_*ns*_ is zero at *q*=1. The minimum value in figure 14*c* occurs at (*p*,*q*)=(1/3,1/2), which coincides with the point at which all non-zero eigenvalues of Δ are equal. The line of equality in figure 14*a* is difficult to express but can be approximated as the straight line *q*=1.0923827*p*−0.3800648, thus for *p*>(*q*+0.3800648)/1.0923827, *V*_*ss*_ will be larger than *V*_*nn*_. In figure 14*b*, the line of equality is given by *q*=(1+3*p*)/4. Likewise, if *q*>(1+3*p*)/4 then *V*_*ns*_<*V*_*sn*_, coinciding with the condition on conductances given in equation (6.7).

## 7. Single-link graphs

**Figure 15.**
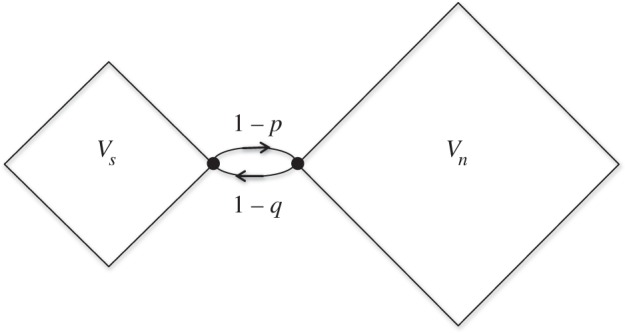
Single line graph.

In this section, we consider graphs consisting of two components with linkage between components occurring only through a single vertex in each component, as illustrated in figure 15.

These graphs and partial bipartite graphs are the extreme cases for graphs having two components with linkages occurring only between subsets of each component. Here the component *V*_*s*_ contains *s* vertices, one of which is linked to one of the *n* vertices in *V*_*n*_. Each non-linked vertex in *V*_*s*_ connect to every other vertex in this component with probability 1/(*s*−1) while the linking vertex connects to all other vertices in *V*_*s*_ with probability *p*/(*s*−1) and connects to the linking vertex in *V*_*n*_ with probability 1−*p*. Likewise, all non-linked vertices in *V*_*n*_ connect to every other vertex in this component with probability 1/(*n*−1) while the linking vertex connects to all other vertices in *V*_*n*_ with probability *q*/(*n*−1) and connects to the linking vertex in *V*_*s*_ with probability 1−*q*.

**Figure 16.**
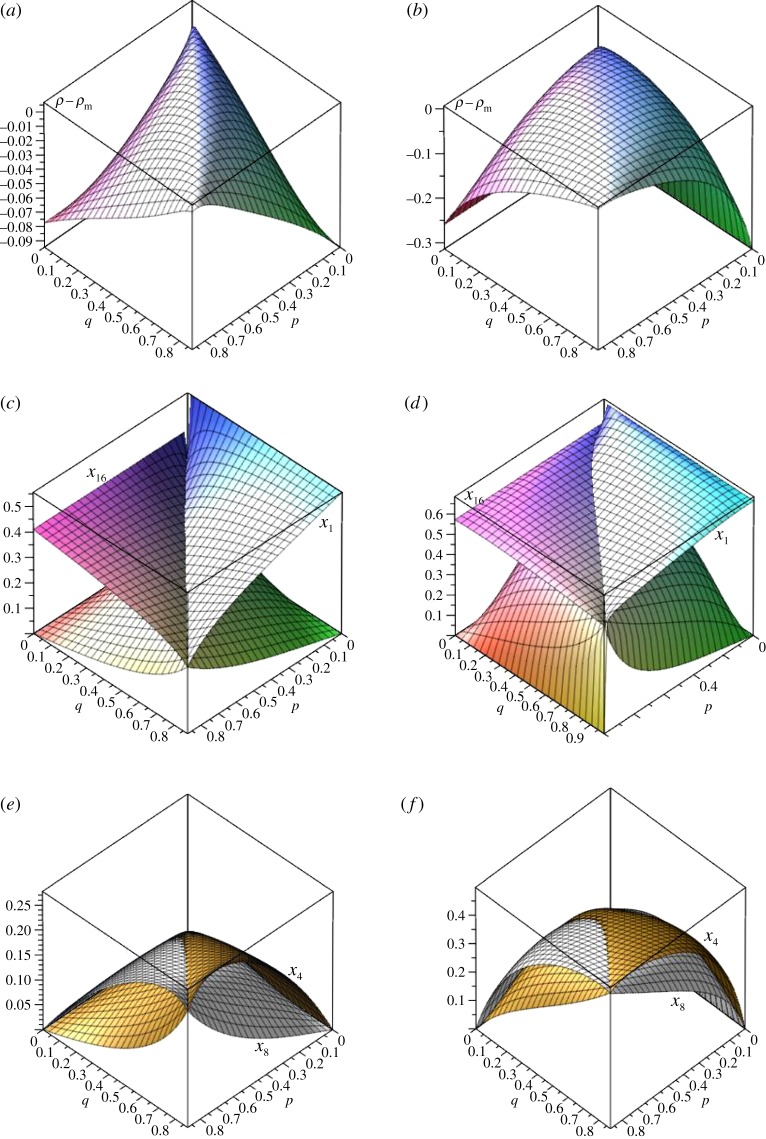
Fixation probabilities for (3,4) single-link graph for *r*=5/4 (*a*,*c*,*e*) and *r*=2 (*b*,*d*,*f*): (*a*,*b*) *ρ*−*ρ*_m_, (*c*,*d*) *x*_1_ and *x*_16_, (*e*,*f*) *x*_4_ and *x*_8_.

Single vertex fixation probabilities are suppressed relative to the Moran probability for cases considered, as shown in figure 16 for (*s*,*n*)=(3,4) for *r*=5/4 and *r*=2. This figure also shows comparisons of fixation probabilities for possible initial mutant vertices for *r*=5/4 and 2. Here *x*_1_ is the fixation probability for a non-linked vertex in *V*_3_, *x*_4_ is the fixation probability for the linking vertex in *V*_3_, *x*_8_ is the fixation probability for the linking vertex in *V*_4_, and *x*_16_ is the fixation probability for a non-linked vertex in *V*_4_.

**Figure 17.**
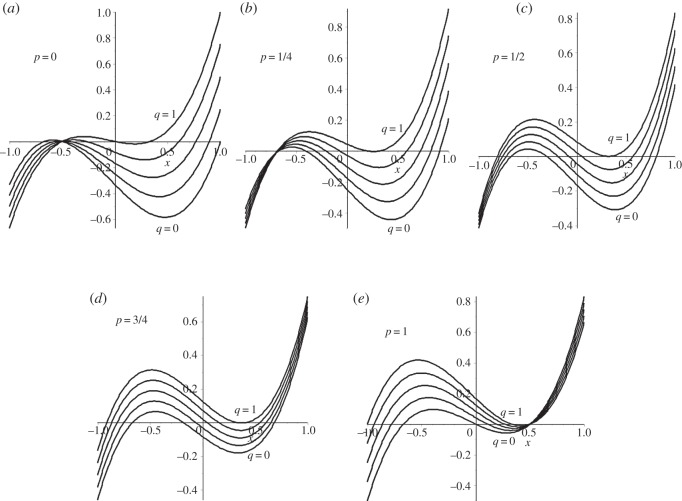
Roots of cubic factor of the (3,4) single-link characteristic polynomial (*a*–*e*) correspond to *p*=0, 1/4, 1/2, 3/4, 1, respectively. In each figure, *q* varies from bottom to top with values 0, 1/4, 1/2, 3/4, 1.

In figure 16*c*,*d*, if *p*=0 the *x*_16_ probability is zero and the *x*_1_ probability is the Moran probability for *s*−1 vertices. Likewise, if *q*=0 the *x*_1_ probability is zero and the *x*_16_ probability is the Moran probability for *n*−1 vertices because fixation can only occur if the initial mutant is introduced in the {*v*_1_,*v*_2_,*v*_3_} or the {*v*_4_,*v*_5_,*v*_6_,*v*_7_} vertex sets, respectively. If *p*=1 (*q*≠1), on the other hand, the *x*_1_ probability is zero and if *q*=1 (*p*≠1), the *x*_16_ probability is zero (these are not shown in figure 16 which only shows *p* and *q* between the values of 0 and 7/8). If *p*=*q*=1 the graph has split into disconnected components, which shows up in the appearance of a second zero eigenvalue for the Laplacian as shows up in figure 17*e* below.

Taking *x*=λ−1, the characteristic polynomial for the Laplacian of this graph is

7.1 $$\begin{array}{rl} & {\left(x-\frac{1}{s-1}\right)}^{s-2}{\left(x-\frac{1}{n-1}\right)}^{n-2}\left\{\left(x^{2}-(1-p)(1-q)\right)\left(x+\frac{s-2}{s-1}\right)\left(x+\frac{n-2}{n-1}\right)\right.\\ & \;-\left.\left[\frac{q}{n-1}\left(x+\frac{s-2}{s-1}\right)+\frac{p}{s-1}\left(x+\frac{n-2}{n-1}\right)\right]x+\frac{pq}{\left(s-1)(n-1\right)}\right\} \end{array}$$

Thus, there will be *s*−2 roots λ=*s*/(*s*−1), *n*−2 roots λ=*n*/(*n*−1). The last term in brackets factors into *x*+1 (yielding the 0 eigenvalue) and a cubic. Figure 17 shows plots of the cubic factor for chosen values of *p* and *q*.

In the lowest curve of figure 17*a*, *p*=*q*=0, corresponding to an eigenvalue of 2 (since *x*=λ−1), whereas in the upper curve of figure 17*e*, *p*=*q*=1, giving a second eigenvalue of 0 (*x*=−1) as the graph is split into two disconnected components.

With the linked vertices denoted *v*_*s*_ and *v*_*n*_, the conductances for the sets *V*_*s*_∖*v*_*s*_, *V*_*n*_∖*v*_*n*_, *v*_*s*_, *v*_*n*_, *V*_*s*_ and *V*_*n*_ are given by

7.2 $$\left.\begin{array}{rl} C\left(\frac{V_{s}}{v_{s}}\right) & =\frac{n+s}{\left(s-1)(n+1\right)},\;C\left(\frac{V_{n}}{v_{n}}\right)=\frac{n+s}{\left(s+1)(n-1\right)}\\ C(v_{s}) & =C(v_{n})=\frac{n+s}{n+s-1}\\ C(V_{s}) & =\frac{\left(n+s)(1-p\right)}{sn}\;\text{and}\;C(V_{n})=\frac{\left(n+s)(1-q\right)}{sn}. \end{array}\right\}$$

All but the two of these are constant and the condition *C*(*V*_*s*_)=*C*(*V*_*n*_) is that *q*=*p*.

**Figure 18.**
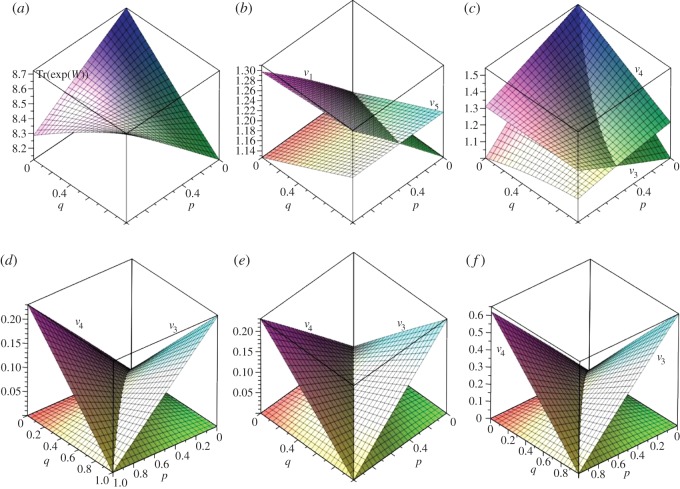
(*a*) Tr(*e*^*W*^/7), (*b*) centrality of *v*_1_ and *v*_5_ vertices, (*c*) centrality of *v*_3_ and *v*_4_ vertices, (*d*) average centrality of *V*_3_ and *V*_4_ components, (*e*) communicability of {*v*_1_,*v*_2_} and {*v*_5_,*v*_6_,*v*_7_} vertex sets, (*f*) communicability of *v*_3_ and *v*_4_ vertices.

Approximation of e^*W*^ and Tr(e^*W*^) for the (3, 4) case yields figure 18.

The minimax values Tr(e^*W*^/7) are at (*p*,*q*)∼(0.793893,0.394234). The line of equality in figure 17*b* is approximately given by *q*∼2.021513*p*+0.0074732—if *q* is greater than the above value, vertex *v*_1_ has the greater centrality. In figure 17*e*, the line of equality is *q*=*p* which, as in the partial bipartite case, corresponds to the condition for *C*(*V*_3_)=*C*(*V*_4_), and we conjecture that this holds for a general single-link graph. Table 3 shows the hitting times for the (3, 4) single-link graph.

**Table 3.** Hitting times for (3, 4) single-linked graph.

$h_{11}=\frac{2+p+2q-5pq}{p(1-q)}$, $h_{33}=\frac{2+p+2q-5pq}{\left(1-q\right)}$,	$h_{55}=\frac{2+p+2q-5pq}{q(1-p)}$ $h_{44}=\frac{2+p+2q-5pq}{\left(1-p\right)}$
$h_{12}=\frac{2(2+p+2q-5pq)}{3p(1-q)}$	$h_{56}=\frac{3(2+p+2q-5pq)}{4q(1-p)}$
*h*_13_=2	$h_{31}=\frac{2(4-p+4q-7pq)}{3p(1-q)}$
$h_{14}=\frac{3}{1-p}$	$h_{41}=\frac{\left(8+p+8q-5pq\right)}{p(1-q)}$
$h_{15}=\frac{3(2+p+2q-3pq)}{2q(1-p)}$	$h_{51}=\frac{2(4+5p+4q-7pq)}{3p(1-q)}$
$h_{34}=\frac{1+2p}{1-p}$	$h_{43}=\frac{1+3q}{1-q}$
$h_{35}=\frac{\left(6+3p+2q-5pq\right)}{2q(1-p)}$	$h_{53}=\frac{4}{1-q}$
$h_{45}=\frac{3(2+p-3pq)}{2q(1-p)}$	*h*_54_=4

In contrast to hitting times for the partial bipartite graphs, most of the hitting times in table 5 show divergences for cases where *p* or *q* are 0 or 1. Consideration of the graph topology makes it apparent that the hitting time *h*_*uv*_ will diverge whenever there is either zero probability of a transition from vertex *u* to vertex *v*, or there is a finite probability of a transition from vertex *u* to a vertex u* for which the probability of transition to vertex *v* is zero.

In terms of symmetries there are natural comparisons between the following pairs of hitting times: (*h*_11_, *h*_55_), (*h*_33_, *h*_44_), (*h*_13_, *h*_54_), (*h*_31_, *h*_45_), (*h*_14_, *h*_53_), (*h*_41_, *h*_35_), (*h*_12_, *h*_56_), (*h*_15_, *h*_51_), (*h*_34_, *h*_43_). Table 4 shows conditions for equality between members of each pair, noting that *h*_13_ can never equal *h*_54_.

**Table 4.** Conditions for equality of pairs of hitting times from table 3.

*h*_11_=*h*_55_, *h*_33_=*h*_44_	*q*=*p*
*h*_31_=*h*_45_, *h*_53_=*h*_14_	$q=\frac{3p}{4-p}$
*h*_12_=*h*_56_	$q=\frac{9p}{8+p}$
*h*_34_=*h*_43_	*q*=3*p*/(4−*p*)
*h*_41_=*h*_35_	$q=\frac{11p^{2}-p+8-\sqrt{76p^{4}-292p^{3}-39p^{2}+272p+64}}{5p^{2}+20p-16}$
*h*_15_=*h*_51_	$q=\frac{-2(4p^{2}+p+4)+\sqrt{73p^{4}-184p^{3}-192p^{2}+320p+64}}{p^{2}-26p+16}$

## 8. Discussion

Construction of graphic population models involves few conceptual problems, the procedure is well established: locate each population member, characterized by one or more variable conditions, at a vertex of a graph and label edges between vertices with some measure of interaction between linked individuals. Then define an updating process relating individual interactions to the dynamics of characteristic variables. While this sort of model can become mathematically complex, the conceptual framework is simple.

Equation (2.1) treats the easiest case, in which each population member exhibits a binary-valued characteristic and the edge weights are just probabilities of interaction, coupled with birth–death updating. Solutions to this equation provide birth–death fixation probabilities for any given initial state.

The problem is the combinatorial explosion that arises. If the population has size *N*, each population member is characterized by a vector of characteristics ***c***=(*c*_1_,…,*c*_*k*_), and each of these characteristics *c*_*i*_ can exhibit one of *m*_*i*_ possible values, then the state space contains almost ${\left(\prod_{i=1}^{k}m_{i}\right)}^{N}$ members.

One way of dealing with this is through methods from statistical mechanics in which graphs are constructed according to algorithms for developing random, small world or other connections between vertices. In this paper, a different direction is taken—we study properties of some very simple graphs in the hope of eventually discovering connections to the statistics of larger graphs. In analogy, a molecular gas can be studied in terms of statistical mechanics by assuming that all molecules are point particles and using concepts such as temperature, entropy, pressure, free energy and so on; but it is also possible to study the structure of the individual molecules with an eye towards eventual discovery of connections between molecular structural properties and large-scale gas behaviour.

Four classes of graphs were considered: biased cycles, dual circular flows, partial bipartite graphs and single-link graphs. For biased cycles, theorem (4.1) gives the characteristic polynomial of the Laplacian matrix and the corollary to this theorem expresses eigenvalues in terms of roots of unity for the special cases *p*=0 and *p*=1. Theorem (4.3) shows the value of the term 1−2*p* as a measure of cycle bias. Computation of hitting times for simple cases shows the characteristic form illustrated in figure 3, with maximum hitting times coming at *p*-values characterizing a ‘tipping’ point between clockwise and counter-clockwise motion for a random walk.

Biased cycles provide the simplest example of the dual circular flow graphs and theorem (5.1) links the characteristic polynomial of the Laplacian of a circular flow to that of the biased cycles, with the term 1−2*p* again playing the role of measuring directional bias. Further generalization allowed the possibility of ‘tuning’ a flow to match specified parameters, exemplified by derivation of the conditions required to match any specified vertex temperature profile.

Bipartite graphs are two-level dual circular flows and, as indicated in the Introduction section, the generalization to partial bipartite graphs leads to results of particular interest, showing a tight connection between conductances, Laplacian eigenvalues, vertex centralities, expected hitting times and single vertex fixation probabilities. These graphs also showed the possibility of counterintuitive behaviour in which fitness values less than 1 can result in enhanced selection, illustrated in figure 11 and table 1. While these tables show only the (2, 5) partial bipartite case, similar behaviour has been found for the (3, 4) case, although the ranges of *p* for which the behaviour appears, at least in preliminary investigation, are narrower. From table 1, for fixed *q* there appears to be two critical *p*-values, *p*_−_ and *p*_+_, depending on *q*, such that in most cases with *q*<1/2 pattern A appears for *p*<*p*_−_, pattern D appears for *p*>*p*_+_ and pattern B for *p*_−_<*p*<*p*_+_; while for *q*≥1/2 pattern C appears rather than pattern B when *p*_−_<*p*<*p*_+_. The value of $r_{\max}$, the fitness value that distinguishes between patterns A and B or between patterns C and D depends on both the *q*- and *p*-values. In table 1, pattern C shows up for cases in which *q* is relatively large while *p* is relatively small. This corresponds to a situation in which the five vertex is strongly connected internally, with weak linkages to the two vertex subset, while this latter subset is weakly connected internally with strong links to the five vertex subset. Under these conditions, a mutant with *r*<1 appearing in *V*_2_ will have a low probability of extinction and, if chosen for reproduction, a high probability of reproducing in *V*_5_ so that in a sense *V*_2_ may act as a root. Much further work is required, however, to develop any detailed understanding of this behaviour, as well as extending analysis to dual circular flows in which vertices in each level can influence each other.

The final case consists of a two-component graph in which the linkage between components occurs only through a single vertex in each component. Laplacian eigenvalues, hitting times and communicability were determined, and fixation probabilities were found for the (3, 4) example, allowing comparison to the corresponding partial bipartite case. As indicated, partial bipartite and single-link graphs are the two extremes of a family of graphs that provide models of two populations communicating across smaller sets of representatives. Adjustment of connection parameters provides models for different levels of polarization in the two populations. Further work is required to analyse both the partial bipartite graphs and single-link graphs, as well as to consider more general cases in which linkages between two populations are through subsets *S*_*k*_⊆*V*_*s*_ and *S*_*m*_⊆*V*_*n*_. Being able to characterize polarization within a population in this way could provide an important tool for studies of social interactions.

Table 5 shows a general summary of results reported in the present paper.

**Table 5.** Summary of results (roman indicates general results, italic indicates limited exemplary cases).

graphs	eigenvalues	conductance	communicability, centrality	expected hitting times	fixation probabilities
biased cycles	theorems (4.1) and (4.2), corollary (4.2), equation (4.2)	*N*/*k*(*N*−*k*) for a length *k* block	*figure 2*	*figure 3a–d*	same as Moran
dual circular flows	theorem (5.1)	*N*/(*N*−*n*_*s*_) for level *s*	*figure 6*	same as biased cycle between levels	*figure 5*
partial bipartite	theorem (6.1), equation (6.5)	equation (6.6)	conjecture (6.2); *figure 14*; equations (6.11) and (6.12);	conjecture (6.2), equations (6.8) and (6.9)	*figures 10–12*, *tables 1 and 2*
single link	*figure 17*, equation (6.15)	equation (6.16)	*figure 18*	tables 3 and 4	*figure 16*
